# A Tamper-Resistant and Portable Healthcare Folder

**DOI:** 10.1155/2008/763534

**Published:** 2008-07-06

**Authors:** Nicolas Anciaux, Morgane Berthelot, Laurent Braconnier, Luc Bouganim, Martine De la Blache, Georges Gardarin, Philippe Kesmarszky, Sophie Lartigue, Jean-François Navarre, Philippe Pucheral, Jean-Jacques Vandewalle, Karine Zeitouni

**Affiliations:** ^1^Institut National de Recherche en Informatique et en Automatique (INRIA), Rocquencourt, 78153 Le Chesnay Cedex, France; ^2^SANTEOS SA, Tour Manhattan, 5,6 Place de l'Iris, 92926 Paris la Défense Cedex, France; ^3^Conseil Général des Yvelines, Hôtel du Département, 2 Place André Mignot, 78012 Versailles Cedex, France; ^4^PRISM Laboratory, University of Versailles, 45 avenue des Etats-Unis, 78035 Versailles Cedex, France; ^5^Association Locale de Développement Sanitaire (ALDS ), 25 avenue des Aulnes, 78250 Meulan, France; ^6^Coordination Gérontologique Intercommunale du Territoire Est Yvelines (CoGITEY), 6 avenue du Maréchal Franchet d'Esperey, 78004 Versailles, France; ^7^Gemalto, 6 rue de la Verrerie, 92190 Meudon, France

## Abstract

Electronic health record (EHR) projects have been launched in most developed countries to increase the quality of healthcare while decreasing its cost. The benefits provided by centralizing the healthcare information in database systems are unquestionable in terms of information quality, availability, and protection against failure. Yet, patients are reluctant to give to a distant server the control over highly sensitive data (e.g., data revealing a severe or shameful disease). This paper capitalizes on a new hardware portable device, associating the security of a smart card to the storage capacity of a USB key, to give back to the patient the control over his medical data. This paper shows how this device can complement a traditional EHR server to (1) protect and share highly sensitive data among trusted parties and (2) provide a seamless access to the data even in disconnected mode. The proposed architecture is experimented in the context of a medicosocial network providing medical care and social services at home for elderly people.

## 1. INTRODUCTION

Since the early days of medicine, and before the advent of computers, people have managed healthcare data manually, accumulating drug prescriptions, examination results, and other medical documents, all of which were inscribed on paper and stored in physical folders at home or at the family doctor office. Although archaic by today's standards, this manual information sharing scheme provided the data owner (i.e., the patient) with control over the sharing and usage of his or her information with the advised assistance of his family doctor and under the protection of the Hippocratic Oath. The patient control was not compromized by the digitization of medical documents in a first stage, simply because the information was scattered among several incompatible information systems in hospitals, clinics, and practitioner's offices. No one knows where the information is, how to access it, whether it is complete and accurate, and under which format it has been produced.

During the last decade, several countries launched ambitious electronic healthcare record (EHR) programs with the objective to increase the quality of healthcare while decreasing its cost [1]. For example, the national *Connecting for Health* (http://www.connectingforhealth.nhs.uk) program in the UK, the *National Switch Point* (https://www.nictiz.nl) managed by Nictiz in the Netherlands, or the healthcare system *Infoway* (http://www. infoway-inforoute.ca/en/home/home.aspx) in Canada are all running projects aiming at building a wide scale EHR. In a recent report [2], French Deputy J.-P. Door identified more than 100 EHR running projects worldwide at the scale of a country or region in a recent report. The objective of centralizing medical information in database systems is manifold (centralization refers to the fact that the data is stored, organized, made available, and controlled by database servers, whatever the computer system infrastructure is): *completeness* (i.e., to make the information complete and up to date), *availability* (to make it accessible through the internet 24 hours/7 days a week), *usability* (to organize the data and make it easily queryable and interpretable), *consistency* (to guarantee integrity constraints and enforce atomicity and isolation of updates), *durability* (to protect the data against failure), and *security* (to protect the data against illegal accesses).

On the other hand, studies in different countries [3, 4], show that several patients and even practitioners are reluctant to use EHR systems arguing increasing threats on individual privacy. This suspicion is fuelled by computer security surveys pointing out the vulnerability of database management systems (DBMSs) against external and internal attacks [5]. Indeed, centralizing and organizing the information make it more valuable, thereby motivating attacks, and facilitates abusive usages. In consequence, EHR providers must comply with very stringent legislation regarding the storage and preservation of medical data. Regardless of the security procedures put in place at the server and their effectiveness, the patient has the sense of losing control over his or her data. There are four main reasons for that.

- *Guidance of the patient consent*: the patient is usually asked to give his consent to an access control policy specifying who (individuals or roles) is granted access to which part of his folder. Even with the help of a practitioner, it is difficult to ensure that this consent is fully enlightened. This is due to the high number of people interacting with the folder, the diversity of their roles, the complexity of the medical information, and the intrinsic difficulty to determine which data (or data association) reveals a given pathology. Consequently, the patient usually adheres to a predefined access control policy that he does not really master. Complementary to access control, audit trails can help the patient tracking a posteriori who accessed which part of his folder and when. However, audit trails exploitation is fairly complex and may require a dedicated query language [6]. With respect to the free expression of the patient consent, EHR systems cannot compete with the archaic manual information sharing scheme.
- *Unbounded data retention*: limited data retention is one of the central principles of laws related to the safeguard of personal data [7]. Limited data retention attaches a lifetime to a data (e.g., 10 years for a given court sentence) after which it must be withdrawn from the system [8]. Unfortunately, limited data retention conflicts with the primary objective of an EHR, that is building a complete medical history of each patient. In addition, [9] highlighted the difficulty to physically destroy information stored in existing DBMSs, showing that it can be recovered by a forensic analysis in many ways. This reinforces the patient's perception that his complete history is recorded forever. A side effect is that the patient may choose not to store some information in his folder (synonym of incompleteness and lower quality of healthcare) because he cannot assess a priori the sensitiveness of this information.
- *No security guarantee outside the server domain*: healthcare data is likely to be extracted from the server and hosted in a client device (e.g., the doctor's or the patient's device). Typically, healthcare folders need to be extracted for use in a disconnected mode, for example, to provide healthcare at home. This situation will remain the rule for a while, that is until every point of the territory be connected through a secure, fast, reliable, and free of charge network. Unfortunately, the hosting device is much more spyware, trojan, and virus prone than the server, introducing an important security breach in the architecture.
- *No disconnected access to the folder:* EHR has been designed with online usage in mind. This may constitute a real barrier for a large category of patients (e.g., elderly, disabled, and needy people), the prerequisite to get access to their folder being either to use a terminal at some public place or to own a PC, to master its use (including the computer administration burden) and to pay for an internet connection. If these latter conditions are not satisfied, a practitioner providing healthcare at home will have to download on his mobile device the folders of all visited patients, a complex and time-consuming task, beside the security breach mentioned above.

In this paper, we propose a novel organization of EHR aiming at circumventing these four drawbacks. This organization capitalizes on a new hardware device called *secure portable token* (*SPT*). Roughly speaking, an SPT combines a secure microcontroller (similar to a smart card chip) with a large external flash memory (gigabyte sized) on a USB key form factor [10, 11]. An SPT can host onboard data and run onboard code with proven security properties thanks to its tamper-resistant hardware and a certified operating system [11]. Embedding a database system and a web server in an SPT gives the opportunity to manage a healthcare folder (or a part of it) outside the EHR server with no loss of security. Accessing the onboard folder while being disconnected from the network requires a simple rendering device equipped with a USB port and running a web browser. More, the embedded DBMS can be made self-administered so that the patient keeps a full control over the onboard data, with no external intervention of a database administrator. The data retention period and the sharing of onboard data can be organized similarly to the previous manual scheme, under the patient control. The resulting architecture is decentralized, with a central server managed by a public or private database service provider and one embedded local server per patient. Splitting the folder in a centralized part and a local part remains under the patient's responsibility. However, the local server cannot provide properties like availability and durability on the local data on its own. We will show that combining the capacities of the central server and the local servers restores these fundamental properties.

Our project is not the first to promote the use of secure tokens. A growing number of initiatives are using smart cards (e.g., the French *Sesam Vitale* [12], the *Patient Health Smart Card* in New York [13], the *National Health Card System,* (24 millions of smart cards, see http://www.gi-de.com/portal/page?_pageid=42,55000&_dad=portal&_schema=PORTAL.), in Taiwan, etc.) to carry patient's national security number and practitioner's certificate in order to implement a strong identification process. Also, the German EHR initiative plans to use smart cards to carry recent prescription information [14]. As our project, the German initiative underlines the interest of holding part of the patient's folder locally. However, this project uses a traditional smart card technology thereby proscribing storing a significant volume of patient's records locally (the smart card is endowed with less than 100 KB of stable memory, (Wiki in German: http://www.de.wikipedia.org/wiki/ Elektronische_Gesundheitskarte), versus 256 MB for the preliminary SPT we are currently working on). To the best of our knowledge, our project is the first to tackle the technical challenges related to the management (storage, querying, and secure sharing) of large folders embedded in secure token.

The paper is organized as follows. Section 2 introduces the functional architecture of the proposed EHR system and discusses different scenarios, showing the benefit of combining a central EHR server with secure portable folders. Section 3 sketches important technical challenges and outlines the solutions proposed. Section 4 describes an application of our solution to a medical network providing healthcare at home and Section 5 concludes.

## 2. FUNCTIONAL ARCHITECTURE AND SCENARIOS

**Figure 1:**
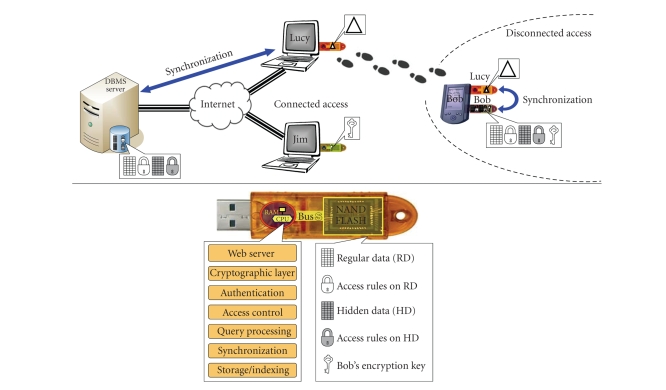
Functional architecture.

The proposed EHR architecture, pictured in Figure 1, is built around a central DBMS server providing the functionalities mentioned above (completeness, availability, usability, consistency, durability, and security) on all patient's folders. This server offers an internet access to all authorized users (e.g., doctors, nurses, etc.) and enforces access control policies defined by the patients. These policies are based on default policies promoted by EHR groups like the GIP DMP (the GIP DMP is in charge of setting up the French medical folder project called DMP (dossier médical personnel). See http://www.d-m-p.org/docs/EnglishVersionDMP.pdf and http://www.d-m-p.org/) and can be refined with the help of practitioners. To allow disconnected accesses to any patient folder, a folder replica is managed on a secure portable token (SPT) provided to each patient. Once endowed with the appropriate embedded software (typically a web server and a DBMS) and timely synchronized with the central server, SPT servers successfully to surrogate the central server. The access control policy defined by the patient is enforced uniformly on the central DBMS and on the SPT. Note that the synchronization between central and SPT servers must occur in all situations (e.g., even if an SPT remains at patient's home and is never connected to the network). We detail this synchronization process later.

SPT is the means by which a patient can recover a full control over his folder, similarly to the manual sharing scheme. Let us assume that a patient is willing to hide information he judges as highly sensitive (e.g., because it may reveal a serious or shameful disease). In a traditional EHR system, if the patient does not fully trust the server or does not fully understand the access control policy (for the reasons mentioned above), he has no other choice than discarding this information, thereby producing an incomplete folder. Here, the patient can store this information, called *hidden data* (*HD*), locally on his SPT without replicating it on the central server. A data which is not hidden is called *regular data* (*RD*) and is replicated on the SPT and the central server. If the patient changes his mind afterwards (e.g., following the advice of his doctor), he still has the opportunity to change the status of a data from hidden to regular. Note that the reverse conversion (from regular to hidden) is uncertain since the data could have been queried and/or copied beforehand. Hiding data matches the privacy objective but the durability property is lost for this data since a portable token is by nature not durable (it might be lost or destroyed). Durability can be restored by using the central server as a repository for cryptoprotected data (i.e., encrypted and signed). To this end, hidden data is encrypted by the patient's SPT and stored encrypted on the central server but the encryption keys are never revealed to the central server. (Note that encryption techniques are sometimes used by central servers to protect the database footprint [15]. With such server-based encryption solutions, data is encrypted and decrypted on the fly by the DBMS. Server-based encryption is thus orthogonal to the SPT-based encryption applied to hidden data). Encryption keys stay under the SPT control and make themselves durable thanks to a trusted depositary. The central server guarantees the durability and availability of hidden data without being able to interpret its content.

Let us now assume that the patient is willing to share hidden data among a restricted *trusted circle* of persons. The patient can define appropriate access control rules on this data so that it becomes accessible to these people in the presence of the patient and of his SPT (as in a paper-based scheme). The SPT allows an even smarter way of exchanging sensitive information. The patient may grant a trusted circle of participants to access his hidden data even if it is not together at the same physical place. This may be helpful in case of emergency or if a remote diagnosis is required. This can be implemented by sharing the encryption keys of the hidden data among the SPTs of people participating in the same trusted circle so that only these SPTs are able to free the hidden data from its cryptoprotection. Note that those keys are never externalized from the SPT, thus allowing enforcing access control rules locally (those rules are stored encrypted along with the hidden data on the central server). To distinguish them in Figure 1, regular (resp., hidden) data and its related access control rules are pictured in white (resp., grey). As mentioned above, the access control policy defined by a patient must be enforced uniformly on the central DBMS and on the SPT, independently of the status of the data. With hidden data, the access control policy is strengthened by the obligation of physically sharing the encryption keys, and this sharing is totally under the patient control. Once decrypted, hidden data is still protected by the access control policy enforced by the SPT.

Let us illustrate the behavior of the system through a scenario involving three participants: an elderly patient named Bob, his family doctor Jim, and a nurse Lucy. Every participant owns an SPT. Several medical examinations are prescribed to Bob who designates a subset of them as hidden (the others being considered as regular). The medical lab performing the examination pushes the results on the central server. Results corresponding to hidden data are cryptoprotected using Bob's public key before being pushed. (Bob's public key is delivered by a PKI server while Bob's private key is replicated on every SPT belonging to Bob's trusted circle. The management of private keys is under the control of the secure chip and even the SPT holder cannot interfere or tamper it. For the sake of conciseness, we do not detail further the key exchange protocol among SPT. For efficiency, asymmetric encryption is used only to encrypt symmetric keys used to protect the hidden data).

Lucy frequently visits Bob at home. Bob has no internet connection and seldom leaves home. Thus, Lucy acts as a synchronization means for Bob's folder (as any other person visiting Bob and owning an SPT). Before the visit, Lucy downloads from the central server only the latest updates, either hidden or regular, performed in Bob's folder. This includes the recent examination results. During the visit, Lucy's and Bob's SPTs are synchronized. The latest updates from the central server are integrated in Bob's local folder. Conversely, the latest updates performed in Bob's local folder, if any, are loaded on Lucy's SPT. This allows refreshing the central server replica the next time Lucy will connect to the server. Synchronized data is only delivered to the recipient (i.e., central server or patient's SPT) using a secure protocol (authentication and encryption). Lucy cannot get access to this data, protected by the tamper resistance of the SPT.

Jim participates in Bob's trusted circle. At his office, he can connect to the central server and view Bob's up-to-date folder, including the results of the recent examinations and possible updates carried back by Lucy (in the limit of Jim's access rights granted by Bob). When visiting Bob at home, Jim can get the same level of information by connecting locally to Bob's SPT.

To summarize, any authorized people (or people playing an authorized role) can connect to the central server or to an SPT local server and retrieve the regular data he is granted access to by the access control policy. No people outside the trusted circle can get access to the hidden data, whatever their role(s) and privilege(s). Indeed, this hidden data is cryptoprotected on the central server and the encryption keys are only known by the patient's SPT and by the SPTs of people belonging to her trusted circle. Hidden data stored on the patient's SPT benefits from the SPT's tamper resistance. Encryption keys (symmetric and private keys) are transmitted from the patient's SPT to the SPTs of the trusted circle using a secure protocol (based on symmetric encryption). This transmission may happen either in a connected mode or via another SPT as part of the synchronization described above. Finally, synchronized data (regular, hidden, or encryption keys) is never disclosed to anyone except the recipient and is also protected during transmission by a secure protocol.

 This mode of operation provides stronger privacy preservation guarantees than any traditional EHR. First, attacks conducted at the server can only reveal regular data, hidden data being absent from the server or encrypted with keys that are let under the control of the clients' SPTs. Most advanced server-based security solutions, even those using encryption [15], cannot offer such a level of protection because encryption keys remain accessible at the server side to enable query execution. Second, attacks conducted at the client terminal cannot reveal more than the data displayed by the application at runtime, but no data is ever stored on client terminals in the clear. Third, the SPT inherits its security from a tamper-resistant hardware and a certified embedded code (certified CC EAL4 or 5, FIPS or using other relevant scheme [11]). We do not argue that SPT is provably unbreakable because ultimate security does not exist but it makes the attacks so complex and costly to implement as they become meaningless in practice. To make the analysis complete, let us consider anyway the impact of an SPT attack. Breaking a patient's SPT will lead to disclose her medical folder stored locally (hidden and regular data); breaking a doctor's SPT will lead to disclose the encryption keys of the patients having registered this doctor in their trusted circle; finally, breaking an SPT serving for synchronization (typically a nurse's SPT) will not disclose any information.

## 3. TECHNICAL CHALLENGES

Many technical challenges are introduced by the proposed EHR architecture, like the access control definition and enforcement, the management of encryption keys, the data synchronization between the central server and the embedded local servers, and so forth. Due to space limitation, this section focuses on the challenges related to the SPT and the embedded data management techniques which are central to the architecture.

### 3.1. SPT hardware and operating system

 An SPT aims at combining in the same hardware platform a secure chip and a mass storage NAND flash memory (several gigabytes soon). The secure chip is of the smart card type, with a 32 bit RISC CPU clocked at about 50 MHz, memory modules composed of ROM, tens of KB of static RAM, a small quantity of internal stable storage (NOR flash) and security modules. A first obvious challenge is to produce this hardware platform and assess its tamper resistance and performance. In this platform, the mass storage NAND flash memory is outside the secure chip (connected by a bus) and does not benefit from the chip hardware protection. A second challenge is then to enforce the integrity and the confidentiality of the data stored in mass storage thanks to the cryptographic capability of the secure chip, with a minimal impact on read/write performance. Considering the increasing storage capacity, computation power and communication throughput of this new generation of smart tokens, integrating them in a distributed architecture as regular computers is a third challenge.

Gemalto, the smart card world leader, is developing an experimental SPT platform. This platform includes a new multitasking operating system allowing the development of web applications based on Java and Servlet technology, and thus offering a standardized means to integrate services or embedded web applications to the SPT. The operating system supports natively:

- the USB 2.0 protocol and the internet protocol IP for communicating with the external world [16];
- multithreaded Java applications;
- cryptographic primitives (some of which being implemented in hardware);
- memory management and garbage collection;
- servlet management and web server.

**Figure 2:**
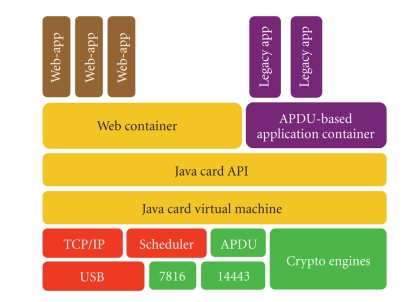
SPT system architecture.

The internal architecture of the SPT in described in Figure 2. For more technical details, we refer the reader to [17].

### 3.2. Embedded database system

DBMS designers have produced light versions of their systems for personal assistants (e.g., Oracle-lite, DB2 everyplace, SQL Server for Window CE) but they never addressed the more complex problem of embedding a DBMS in a chip. Initial attempts toward a smart card DBMS were ISOL's SQL Java machine [18], the ISO standard SCQL [19], and the MasterCard Open Data Storage [20]. All these proposals concerned traditional smart cards with few resources and therefore proposed basic data management functionalities (close to sequential files). Managing embedded medical folders requires much more powerful storage, indexation, access control, and query capabilities. PicoDBMS was the first full-fledged relational DBMS embedded in a smart card [21] and was implemented on top of Gemalto's smart card prototypes [22]. The peculiarities of secure chip environments compel to deeply revisit existing DBMS techniques like storage and indexing models, query execution strategies and transaction management. PicoDBMS has been designed for managing databases stored in a (megabyte sized) EEPROM stable memory integrated in the secure chip and protected by the chip tamper resistance.

The SPT framework introduces important new challenges [23].

1. How to support complex access rights and queries over a gigabyte-sized onboard database (compared to PicoDBMS, the database size grows by three orders of magnitude while the RAM resource was multiplied roughly by 5)?
2. How to organize the data storage and the indexes with an acceptable insert/update time considering the peculiarities of NAND flash memory (fast reads, costly writes, block-erase-before-page-rewrite constraint)?
3. How to protect the onboard database against confidentiality and integrity attacks without degrading the query performance; this challenge is related to the flash protection problem mentioned in Section 3.1 but solutions specific to database management can be devised to provide optimal performance?

**Figure 3:**
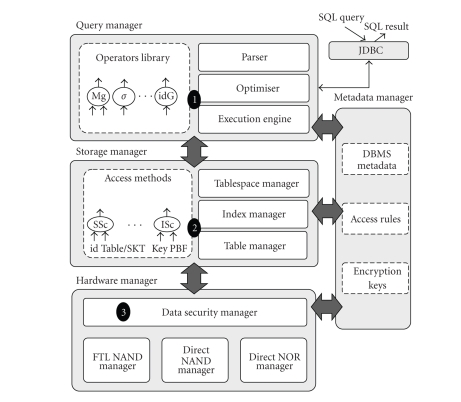
DBMS kernel architecture.

Figure 3 depicts the main software modules of the embedded DBMS, tagged with numbers indicating the related technical challenge.

The *Query Manager* is in charge of parsing the incoming SQL query, building an optimal query execution plan, and executing it. This module must consider peculiar execution strategies to answer complex SQL queries over a large quantity of data (gigabytes) while coping with the SPT hardware constraints (challenge 1). To tackle this challenge, we designed a massive indexing scheme presented in [24], which allows processing complex queries while consuming as little RAM as possible and still exhibiting acceptable performances. The idea is to combine in the same indexing model generalized join indices and multitable selection indices in such a way that any combination of selection and join predicates can be evaluated by set operations over lists of sorted tuple identifiers. The operator library (algorithms for the operators of the relational algebra, e.g., select, project, join, and aggregate) and the execution engine integrate those techniques.

The *Storage Manager*, on which the query manager relies to access the database content (index and tables), is directly concerned with challenge 2. Indeed, the proposed massive indexation scheme causes a difficult problem in terms of flash updates, due to the severe read/write constraints of NAND flash. Therefore, we designed a structure which manages data and index keys sequentially so that the number of rewrites in flash is minimized. The use of summarization structures (based on bloom filters [25]) and vertical partitioning reduce the cost of index lookups. These additional structures are also managed in sequence. A first implementation of this principle has been patented jointly by INRIA and Gemalto [26] and is integrated in the current DBMS prototype.

The *Hardware Manager* embeds the methods for accessing the different memory modules of the SPT (through the Flash Translation Layer (FTL) [27] or with direct access). It includes techniques associated with challenge 3, to protect the confidentiality and the integrity of the data, in an efficient way with respect to DBMS access patterns. Indeed, our massive indexation technique leads to numerous, random, and fine grain accesses to raw data. We conducted preliminary studies [28], in which we combine encryption, hashing, and timestamping techniques with query execution techniques in order to satisfy three conflicting objectives: efficiency, high security, and compliance with the chip hardware resources.

Finally, the *Metadata Manager* manages the DBMS metadata, the access control rules regulating the access to regular and hidden data and the encryption keys.

## 4. EXPERIMENTAL PLATFORM

### 4.1. Experiment in the field

The functional architecture presented in Section 2 will be experimented in the context of a medicosocial network providing medical care and social services at home for elderly people in the Yvelines district, France. Today, the coordination among the participants of this network (doctors, nurses, physiotherapists, social workers, etc.) is organized around a paper-based folder. This folder stays at home and is queried and updated by every participant. This solution suffers from two main drawbacks. First, instead of providing a natural and controlled way of sharing medical and social information, the paper-based folder is the primary source of confidentiality breach in this setting. Indeed, this folder must be shared by all participants but there is no means by which an effective access control policy can be implemented. Second, the folder cannot be accessed and filled remotely, precluding remote diagnosis, and is often incomplete due to its physical centralization. Replacing this paper-based folder system by a traditional EHR would introduce the drawbacks mentioned in Section 1. The proposed experimentation will combine a central database with medicosocial folders embedded in SPT, according to the architecture presented in the previous section.

This experimentation will be conducted with a population of about 100 volunteer patients and 25 practitioners and social workers in the Yvelines district in France. It involves the following partners: the French National Research Institute in Computer Sciences (INRIA), University of Versailles, SANTEOS (EHR provider for the prefiguration phase of the French national healthcare folder system), Gemalto (world leader in the smart card domain), ALDS (a home healthcare association), and CoGITEY (a clinic with a section dedicated to elderly people). This project is partly funded by the Yvelines district council and by the French National Agency for Research (ANR). The design phase started in January 2007 and the experimentation in the field will be conducted fall 2009.

### 4.2. Software platform

**Figure 4:**
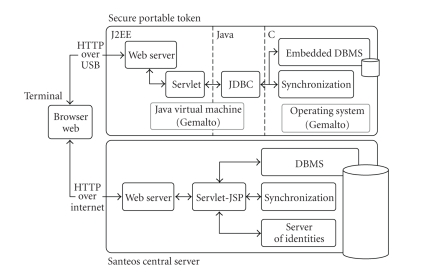
Software platform.

In the experiment, the final user (either practitioner or patient) will be able to connect to a server (either central or embedded) with a web browser running on any terminal (fixed or mobile). A web-based interface (GUI) is provided to browse the patient folder. By manipulating the GUI, the user generates HTTP requests to the server, thereby activating Servlets which in turn generate database queries and build the next page of the interface. Whatever the server it is connected to, the GUI provides similar functions, for example, access to patients' folders, authorization management, and so forth. The software platform enabling this behavior is presented in Figure 4.

The central server is equipped with commercial software including a web server (the GUI is generated using Servlets and JavaServer Pages), a relational DBMS (to store, index, and retrieve patients' folders), and a server of identities (to manage certificates and identifiers for medical and social workers and patients).

The SPT embeds a proprietary web server and Servlets communicating with a lightweight DBMS engine via a JDBC-like driver. A synchronization module is also embedded in the SPT to synchronize the embedded folder with the copy stored in the central server. Thus, the software deployed on the central server and the SPT provide similar functionalities while relying on highly different technology to cope with the SPT hardware constraints. For more information about the software platform, we refer the reader to [17].

## 5. CONCLUSION

In this paper, we presented an alternative to centralized EHR systems, relying on a new hardware portable device called *secure portable token* (*SPT*). This architecture is being implemented in the scope of the DMSP and PlugDB projects started in November 2006 and will be experimented in the fall of 2009 in the context of a medical social network providing medical care and social services at home for elderly people.

The objectives pursued are

- to build a shared medicosocial folder providing the highest degree of availability, whatever the mode of operation (disconnected or not);
- to reestablish a natural and powerful way of protecting and sharing highly sensitive information among trusted parties.

The expected outcome of this project is to demonstrate that these two objectives can be reached with a positive impact on the coordination of medical and social workers and on the acceptation of patients of an electronic usage of their medical history.
